# Monovalent Ions and Water Dipoles in Contact with Dipolar Zwitterionic Lipid Headgroups-Theory and MD Simulations

**DOI:** 10.3390/ijms14022846

**Published:** 2013-01-29

**Authors:** Aljaž Velikonja, Šarka Perutkova, Ekaterina Gongadze, Peter Kramar, Andraž Polak, Alenka Maček-Lebar, Aleš Iglič

**Affiliations:** 1 Laboratory of Biocybernetics, Faculty of Electrical Engineering, University of Ljubljana, Tržaška 25, SI-1000 Ljubljana, Slovenia; E-Mails: aljaz.velikonja@avelik.homeip.net (A.V.); peter.kramar@fe.uni-lj.si (P.K.); andraz.polak@fe.uni-lj.si (A.P.); alenka.macek-lebar@fe.uni-lj.si (A.M.-L.); 2 SMARTEH Research and Development of Electronic Controlling and Regulating Systems, Trg tigrovcev 1, SI-5220 Tolmin, Slovenia; 3 Laboratory of Biophysics, Faculty of Electrical Engineering, Tržaška 25, University of Ljubljana, SI-1000 Ljubljana, Slovenia; E-Mails: sarka.perutkova@fe.uni-lj.si (S.P.); ekaterina.gongadze@fe.uni-lj.si (E.G.); 4 Laboratory of Clinical Biophysics, Orthopaedic Clinics and Faculty of Medicine, University of Ljubljana, Vrazov trg 2, SI-1000 Ljubljana, Slovenia

**Keywords:** lipids, dipolar zwitterionic headgroups, relative permittivity, orientational ordering, water molecules, planar lipid bilayers

## Abstract

The lipid bilayer is a basic building block of biological membranes and can be pictured as a barrier separating two compartments filled with electrolyte solution. Artificial planar lipid bilayers are therefore commonly used as model systems to study the physical and electrical properties of the cell membranes in contact with electrolyte solution. Among them the glycerol-based polar phospholipids which have dipolar, but electrically neutral head groups, are most frequently used in formation of artificial lipid bilayers. In this work the electrical properties of the lipid layer composed of zwitterionic lipids with non-zero dipole moments are studied theoretically. In the model, the zwitterionic lipid bilayer is assumed to be in contact with aqueous solution of monovalent salt ions. The orientational ordering of water, resulting in spatial variation of permittivity, is explicitly taken into account. It is shown that due to saturation effect in orientational ordering of water dipoles the relative permittivity in the zwitterionic headgroup region is decreased, while the corresponding electric potential becomes strongly negative. Some of the predictions of the presented mean-field theoretical consideration are critically evaluated using the results of molecular dynamics (MD) simulation.

## 1. Introduction

The lipid bilayer is a basic building block of cell membranes. Although the cell membrane is a highly heterogeneous structure, composed of lipids, proteins, carbohydrates and other components [1–3], a large number of physical properties of biological (cell) membrane which are important in physiological processes [4,5] can be studied also in pure planar lipid bilayer systems [6,7]. Due to its double-sided accessability in experiments the planar lipid bilayer is suitable for experimental manipulation [8,9] even if some other pure lipids systems, like lipid vesicles, may mimic also three-dimensional geometry of the cell membrane.

**Figure 1:**
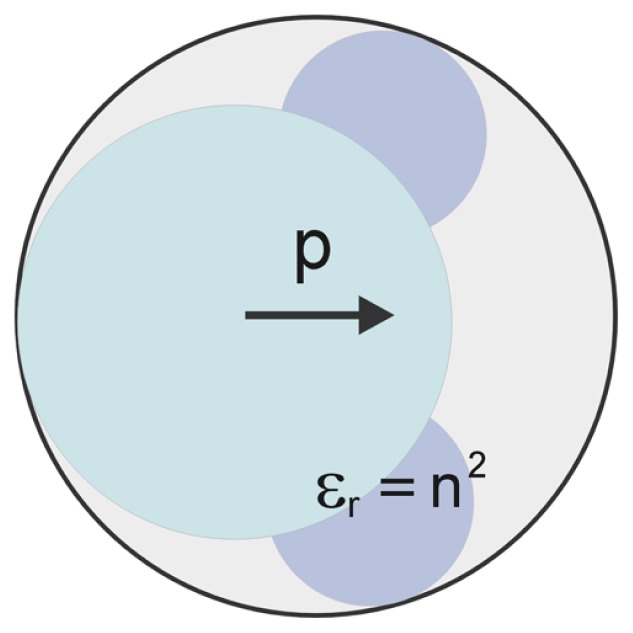
A single water molecule is considered as a sphere with permittivity *n*^2^ and point-like rigid (permanent) dipole with dipole moment **p** at the center of the sphere [13], where *n* is the optical refractive index of water.

Among others a planar lipid bilayer can be constructed on a small hole positioned on a barrier separating two compartments filled with electrolyte solution. Experimental setup usually consists of two teflon pieces with separate compartments and 100 *μ*m–1mm diameter hole in between. The diameter of the hole mostly depends on the procedure used in planar lipid bilayer formation [10]. In experiments, the electrodes are dipped into electrolyte solution approximately 1 cm away form the barrier. Electrolyte solution is basically an aqueous solution of salt composed of water molecules (Figure 1) and monovalent positively and negatively charged ions. Such experimental setup is ordinarily used to determine various mechanical and electrical properties of planar lipid bilayers [9,11,12].

Most frequently used lipid molecules in the procedures of forming the planar lipid bilayers are glycerol-based phospholipids [6,7,14], which are major compound of cell membranes [15–18]: 1-palmitoy*l*-2-oleoyl-sn-glycero-3-phosphocholine (POPC), 1-palmitoyl-2-oleoyl-sn-glycero-3-phospho-L-serine (POPS), 1,2-dipalmitoyl-sn-glycero-3-phosphocholine (DPPC), L-*α*-phosphatidylcholine (PC), 1-palmitoyl-2-oleoyl-sn-glycero-3-phosphoethanolamine (POPE), 1,2-dipalmitoyl-sn-glycero-3-phosphoethanolamine (DPPE), and 1,2-dimyristo-yl(d54)-sn-glycero-3-phosphocholine (DMPC). The majority of these lipids can be made artificially, *i.e*., without need to isolate them from natural cell membranes. Artificially made lipids are more than 99 percent pure, and easy to handle. They are available in powder state form or dissolved in chloroform [9].

**Figure 2:**
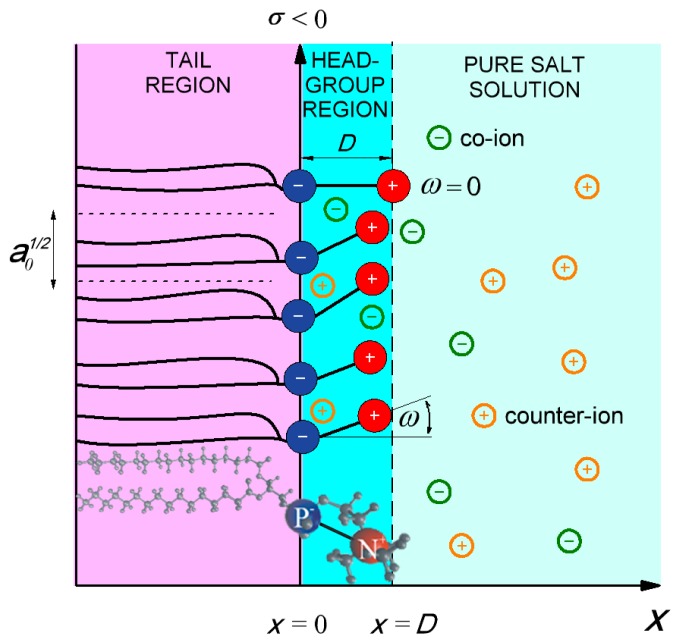
Negative charges of dipolar (zwitterionic) lipid headgroups are described by negative surface charge density *σ* = −*e*_0_*/a*_0_ at *x* = 0, where *a*_0_ is the area per lipid. *D* is the distance between charges within the single dipolar lipid headgroup, while *ω* describes orientation angle of the dipole within the single headgroup. MD model of DPPC lipid molecule is presented at the bottom.

In general, some of the lipid molecules bear net negative charge, while other lipids like glycerophospholipids are electrically neutral [14]. Glycerophospholipids (see Figure 2) are composed of dipolar (zwitterionic) headgroup and two nonpolar tails [1,2,6,7,14]. The tails are hydrophobic fatty acids. Due to its hydrophilic nature [14] the headgroups of lipids are in contact with aqueous solution. The negative charges of the dipolar lipid headgroup (Figure 2) are in contact with nonpolar tails on the one side (left side in Figure 2) and with electrolyte solution on the other (right side in Figure 2), thus its charge distribution is described in the model as negatively charged surface at *x* = 0 (Figure 2). The positive charges of the headgroups in planar bilayer of dipolar lipids protrude further in the electrolyte solution as depicted in Figure 2. In the headgroup region of planar dipolar lipid layer the cations are attracted towards the negatively charged surface at *x* = 0, while anions are depleted from this region. Using the MD simulations, the accumulation of sodium cations near the phosphate groups and accumulation of anions near the choline groups in the DOPC bilayer were predicted by [19]. These theoretical predictions were supported also by the results of fluorescence spectroscopy experiments on zwitterionic phospholipid bilayers which show that the addition of salt into the solution being in contact with the lipid bilayer restricts the mobility of the hydrated lipid headgroups and their lateral diffusion when compared to pure water solution [19]. In the high electric field of dipolar lipid headgroups (see [20] and the references therein) the water dipoles are expected to be oriented towards the charged plane at *x* = 0 (see for example [13,21]). Due to accumulation of cations and saturation effect in polarization the electric potential decreases towards the *x* = 0 plane [22].

Most of the electrostatic models of electrolyte solution in contact with lipid surfaces [20,23–26] assume that the dielectric permittivity in the electrolyte solution is constant. In the absence of an explicit consideration of orientational ordering of water molecules the assumption of constant permittivity is the consequence of the assumption of the constant number density of water molecules in the system [13]. But actually, close to the membrane surface the orientation and depletion of water molecules may result in strong spatial variation of permittivity [13,27–31].

In this paper the effect of the nonhomogeneous volume charge distribution in the headgroup region of the planar dipolar lipid layer on the space dependent electric potential and permittivity is presented. An analytical mean-field model, based on the previously developed Langevin-Poisson-Boltzman (LPB) model [21], is introduced. The relative (dielectric) permittivity, related to the electric field strength was analysed in the headgroup region and its close vicinity. To test the predictions of the model, the realistic values of the input model parameters, previously determined in measurements or simulations for DPPC lipid molecules, were used. The results of an analytical model are compared with the results of molecular dynamic simulation (MD) of DPPC planar lipid bilayer.

## 2. Model

### 2.1. Modified Langevin-Poisson-Boltzmann (MLPB) Model

The Langevin-Poisson-Boltzmann (LPB) model [21] is generalized to take into account the cavity field [13] in the saturation regime. In addition, the electronic polarization of the water is taken into account by assuming that the point-like rigid (permanent) dipole embedded in the center of the sphere with a volume equal to the average volume of a water molecule in the electrolyte solution (Figure 1) [13,32]. The permittivity of the sphere is taken to be *n*^2^, where *n* = 1.33 is the optical refractive index of water. The relative (effective) permittivity of the electrolyte solution *ɛ_r_* can be then expressed as [13,32] :

(1) $${ɛ}_{r}(x)=n^{2}+\frac{P(x)}{{ɛ}_{0}E(x)}$$

where *P* = *|***P***|* is the magnitude of the polarization vector due to a net orientation of permanent point-like water dipoles having dipole moment **p**, *ɛ*_0_ is the permittivity of the free space, while *E* = *|***E***|* is the magnitude of the electric field strength. The absolute value of polarization *P*(*x*) is given by [13] :

(2) $$P(x)=n_{w}(x)\;\left(\frac{2+n^{2}}{3}\right)\;p_{0}L(\gamma p_{0}E\beta )$$

where *n_w_*(*x*) is the space dependency of the number density of water molecules, *p*_0_ is the magnitude of the external dipole moment **p***_e_* = (3*/*(2 + *n*^2^)) **p** (see also Figure 1) [13,32], *ℒ*(*u*) = (coth(*u*)−1*/u*) is the Langevin function, *β* = 1*/kT*, *kT* is thermal energy, while *γ* is [13] :

(3) $$\gamma =\frac{3}{2}\left(\frac{2+n^{2}}{3}\right)$$

In the following, for the sake of simplicity the finite volume of ions and water molecules is not taken into account as in [13] and consequently the number density of water molecules is considered to be constant all over the solution and equal to its bulk value *n_w_*_0_, *i.e*., *n_w_*(*x*) = *n*_0_*_w_* from where it follows :

(4) $$P(x)=n_{0w}\;\left(\frac{2+n^{2}}{3}\right)\;p_{0}L(\gamma p_{0}E\beta )$$

Combining Equations 1 and 4 yields space dependency of permittivity within modified Langevin-Poisson-Boltzmann model (MLPB model) in the form:

(5) $${ɛ}_{r}(x)=n^{2}+\frac{n_{0w}\;p_{0}}{{ɛ}_{0}}\left(\frac{2+n^{2}}{3}\right)\frac{L(\gamma p_{0}E(x)\beta )}{E(x)}$$

or

(6) $${ɛ}_{r}(x)=n^{2}+\frac{3}{2}{\left(\frac{2+n^{2}}{3}\right)}^{2}\frac{n_{0w}{p_{0}}^{2}\beta }{{ɛ}_{0}}\frac{L(\gamma p_{0}E(x)\beta )}{\gamma p_{0}E(x)\beta }$$

In the limit of vanishing electric field strength (*E*(*x*) → 0 everywhere in the solution) the above equation for relative permittivity *ɛ_r_*(*x*) gives the classical Onsager expression:

(7) $${ɛ}_{x}≅n^{2}+{\left(\frac{2+n^{2}}{3}\right)}^{2}\frac{n_{0w}{p_{0}}^{2}\beta }{2{ɛ}_{0}}$$

At room temperature (298K) the above Equation 7 gives *ɛ_r_* = 78.5 for bulk solution. The parameters *p*_0_ and *n*_0_*_w_/N_A_* are 3.1 Debye and 55 mol*/*l, respectively.

### 2.2. Poisson Equation

In the model the dipolar lipid headgroup is described by two charges at fixed distance *D*, *i.e*., it is assumed that the headgroup has non-zero dipole moment. The negative charges of the phosphate groups of dipolar (zwitterionic) lipids are described by negative surface charge density *σ* = −*e*_0_*/a*_0_ at *x* = 0 (see Figure 2), where *a*_0_ is the area per lipid. The orientational ordering of water is taken into account assuming the spatial dependence of permittivity *ɛ_r_*(*x*) as described by Equation 6.

**Figure 3:**
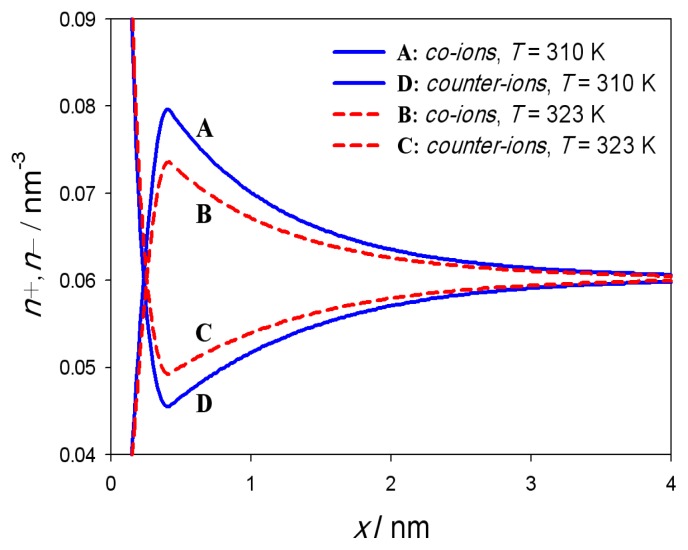
The calculated charge density profile of co-ions (*n*_−_) (A,B) and counter-ions (*n*_+_) (C,D) of the electrolyte solution for two temperatures 310K (full blue line) and 323K (dashed red line) and corresponding DPPC values of the area per lipid (*a*_0_ = 0.48nm^2^ below 314K and *a*_0_ = 0.60nm^2^ above 314K). The dipole moment of water was *p*_0_ = 3.1 Debye, *D* = 0.42nm, bulk concentration of salt *n*_0_*/N_A_* = 0.1 mol*/*l and concentration of water *n*_0_*_w_/N_A_* = 55 mol*/*l, where *N_A_* is Avogadro number.

The corresponding Poisson equation in planar geometry can thus be written in the form (see e.g., [22]) :

(8) $$\frac{\text{d}}{\text{d}x}\left[{ɛ}_{0}{ɛ}_{r}(x)\frac{\text{d}\varphi }{\text{d}x}\right]=-\rho_{ions}(x)-\rho_{Zw}(x)$$

where *φ*(*x*) is the electric potential, *ρ_Zw_*(*x*) is the macroscopic (net) volume charge density of positive charges of dipolar (zwitterionic) headgroups, *ρ_ions_*(*x*) is the macroscopic (net) volume charge density of co-ions (*n*_−_) and counter-ions (*n*_+_) of the electrolyte solution (see Figure 3). Since we neglect the finite volumes of the salt ions and water molecules [21,22] the co-ions and counter-ions are assumed to be distributed according to Boltzmann distribution functions [20,22–26]) :

(9) $$n_{+}(x)=n_{0}\;\text{exp}(-e_{0}\varphi (x)\beta )$$

(10) $$n_{-}(x)=n_{0}\;\text{exp}(e_{0}\varphi (x)\beta )$$

therefore

(11) $$\rho_{ions}(x)=e_{0}\;n_{+}(x)-e_{0}\;n_{-}(x)=-2\;e_{0}\;n_{0}\;\text{sinh }e_{0}\varphi \beta$$

where *e*_0_ is the unit charge and *n*_0_ bulk number density of salt co-ions and counter-ions. The lipid headgroups can be oriented at various angles *ω* relative to the normal vector to the planar lipid layer (Figure 2), hence the volume charge density due to the positive charges of the lipid dipolar headgroups can be written in the form:

(12) $$\rho_{Zw}(0<x\leq D)=\frac{e_{0}P(x)}{D\;a_{0}}\;\;\;\text{and }\;\;\rho_{Zw}(x>D)=0$$

where *a*_0_ is the area per lipid, while *℘*(*x*) is the probability density function indicating the probability that the positive charge of the dipolar lipid headgroup is located at the distance *x* from the negatively charged surface at *x* = 0 :

(13) $$P(x)=\Lambda \;\text{exp}(-e_{0}\varphi (x)\beta )$$

where *x* ≤ *D*. Equation 13 neglects the finite volume of lipid headgroups. The normalization condition

(14) $$\frac{1}{D}\int_{0}^{D}P(x)\;\text{d}x=1$$

yields :

(15) $$\Lambda =\frac{D}{\int_{0}^{D}\text{exp}(-e_{0}\varphi (x)\beta )\;\text{d}x}$$

Using Equations 11–13 and 15 it follows from Equation 8 :

(16) $$\frac{\text{d}}{\text{d}x}\left[{ɛ}_{0}\;{ɛ}_{r}(x)\frac{\text{d}\varphi }{\text{d}x}\right]=2\;e_{0}\;n_{0}\;\text{sinh }e_{0}\varphi \beta -\frac{e_{0}\;\text{exp}(-e_{0}\varphi (x)\beta )}{a_{0}\int_{0}^{D}\text{exp}(-e_{0}\varphi (x)\beta )\;\text{d}x}$$

The boundary conditions are (see for example [22]) :

(17) $$\frac{\text{d}\varphi }{\text{d}x}(x=0)=-\frac{\sigma }{{ɛ}_{0}\;{ɛ}_{r}(x=0)}$$

(18) $$\frac{\text{d}\varphi }{\text{d}x}(x\to \infty )=0$$

(19) $$\varphi (x=D_{-})=\varphi (x=D_{+})$$

(20) $$\frac{\text{d}\varphi }{\text{d}x}(x=D_{-})=\frac{\text{d}\varphi }{\text{d}x}(x=D_{+})$$

where in Equation 17 the surface charge density *σ* = −*e*_0_*/a*_0_. Note that the area per lipid *a*_0_ is different in gel and liquid phase. *E* = *|φ*′*|*.

In numerical calculations the distance from the negatively charged surface *x* was limited to 12nm, where the boundary condition stated in Equation 18 was applied. The modified LPB equation (Equation 16) was solved numerically as described in the Appendix A. All the results were obtained using *a*_0_ = 0.48nm^2^ and *a*_0_ = 0.60nm^2^ corresponding to DPPC lipid in gel-crystalline (below 314K) and liquid-crystalline phase (above 314K), respectively [33]. Other values of model parameters were: the dipole moment of water *p*_0_ = 3.1 Debye, bulk concentration of salt *n*_0_*/N_A_* = 0.1 mol*/*l and concentration of water *n*_0_*_w_/N_A_* = 55 mol*/*l. *N_A_* is Avogadro number.

### 2.3. Molecular Dynamics Simulations (MD)

The molecular dynamics (MD) model of DPPC planar lipid bilayer was constructed in NAMD program using all molecule performance CHARMM 36 force field. The model consists of 256 lipid units and 20174 water molecules. The solvent was 450mMKCl modeled by 153K^+^ and 153 Cl^−^ ions [34,35]. Chemical bonds between hydrogen and heavy atoms were constrained to their equilibrium value. Long-range electrostatic forces were taken into account using a fast implementation of the particle mesh Ewald (PME) method [36,37]. The model was examined at constant pressure (1.013 *×* 10^5^ Pa) and constant temperature (232K) employing Langevin dynamics and the Langevin piston method. The equations of motion were integrated using the multiple time-step algorithm. A time step of 2.0 fs was employed. Short- and long-range forces were calculated every one and two time steps, respectively.

The model was equilibrated and followed 30 ns. The last 15 ns of the simulation were used for extraction of dipole orientation angle. From the P and N atoms positions the dipole was determined for all 256 lipids in each of 1500 simulation frames and exported to Matlab2012b. The distribution of vector amplitude corresponding to distance *D* between charges was extracted as well as distribution of the angle *ω* between the dipole and normal vector to the planar lipid bilayer plane (Figure 2). To obtain the probability density *℘*(*x*), projection of each headgroup dipole on the normal vector to the planar lipid bilayer plane was calculated. The average distance between P and N atoms (0.42nm) was used as a parameter *D* in MLPB model (Equation 16).

## 3. Results

**Figure 4:**
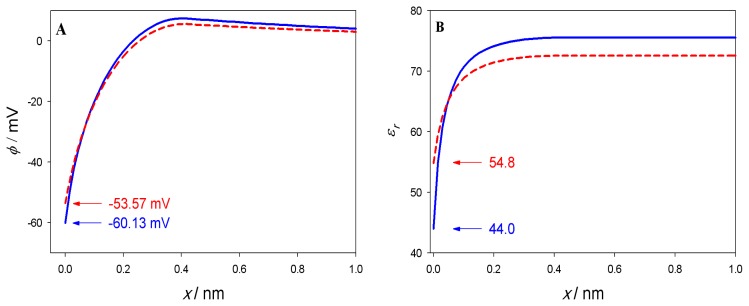
Electric potential *φ* and relative permittivity *ɛ_r_* as a function of the distance from the charged planar surface at *x* = 0 calculated for two temperatures and corresponding values of *a*_0_: *T* = 310K, *a*_0_ = 0.48nm^2^ (full blue lines) and *T* = 323K, *a*_0_ = 0.60nm^2^ (dashed red lines). These values of *a*_0_ correspond to DPPC in two different phases. Relative permittivity *ɛ_r_* as the function of the distance from the charged planar surface *x* (panel B) was calculated from Equation 6. The MLPB equation was solved numerically as described in the Appendix A. The dipole moment of water *p*_0_ = 3.1 Debye, *D* = 0.42nm bulk concentration of salt *n*_0_*/N_A_* = 0.1 mol*/*l, concentration of water *n*_0_*_w_/N_A_* = 55 mol*/*l, where *N_A_* is Avogadro number. For simplicity the second boundary condition was applied at a distance of 12nm.

Electric potential *φ* and relative permittivity *ɛ_r_* as a function of the distance from the charged planar surface (*x* = 0) is presented in Figure 4. The results are presented for two values of the temperature: *T* = 310K (*a*_0_ = 0.48nm^2^) and *T* = 323K (*a*_0_ = 0.60nm^2^). It can be seen in Figure 4 that the relative permittivity *ɛ_r_*(*x*) is considerably decreased in the vicinity of charged planar surface. At the charged planar surface (*x* = 0) the value of *ɛ_r_*(*x*) drops to 44 at *T* = 310K and to 55 at *T* = 323K. The effect of the charged planar surface at *x* = 0 is very weak already at the distance *x* = *D*. Far away from the surface (*x* = 0) the values of *ɛ_r_*(*x*) is 75.5 at temperature *T* = 310K and 72.6 for temperature *T* = 323K. The electric potential in the vicinity of the charged planar surface is considerably negative. It is −60mV for temperature *T* = 310K and −54mV for temperature *T* = 323K.

Charge density profile of co-ions (*n*_−_) and counter-ions (*n*_+_) of the electrolyte solution for two temperatures 310K and 323K can be seen in Figure 3. Near the negatively charged planar surface at *x* = 0 one can observe strong accumulation of positively charged counter-ions and depletion of the negatively charged co-ions. With increasing distance from the headgroup region (0 *< x* ≤ *D*), *i.e*., for *x* larger than *D*, the number density of co-ions (*n*_−_) decreases and the number density of counter-ions (*n*_+_) increases. Far away from the charged planar surface, the concentration of counter-ions (*n*_+_) equals the concentration of co-ions (*n*_−_) corresponding to electroneutrality condition in bulk solution. At higher temperature (323K) the DPPC has increased area per lipid *a*_0_ = 0.60nm^2^ resulting in lower area density of the lipid molecules and hence less negative surface charge density at *x* = 0 plane as in the case of lower temperature (310K). Consequently, also the calculated ion number density profiles are lower for *x > D* (Figure 3).

**Figure 5:**
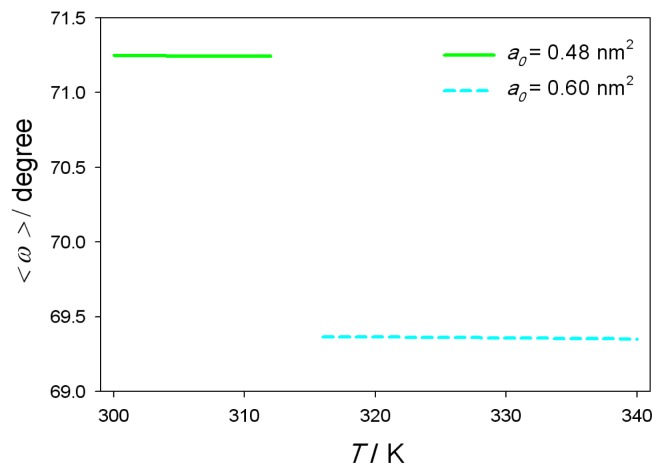
Average headgroup dipole orientation angle *< ω >* (see also Figure 2) as a function of the temperature *T*. Area per lipid *a*_0_ = 0.48nm^2^ below 314K, corresponding to DPPC lipid gel-crystalline phase (full line) and *a*_0_ = 0.60nm^2^ above 314K, corresponding to DPPC lipid liquid-crystalline phase (dashed line). A gap near the phase transition temperature (314K) is present, because phase transition effect is not included in MLPB model. Average dipole orientation angle *< ω >* was calculated from *℘* (*x*) as described in Appendix B. The values of other model parameters are the same as in Figure 4.

The average headgroup orientation angle *< ω >* as a function of the temperature *T* can be seen on Figure 5. At DPPC phase transition temperature (314K) the value of *< ω >* can not be calculated as phase transition effect is not included in MLPB model. The average dipole orientation angle *< ω >*, calculated from *℘*(*x*) as described in Appendix B, is not temperature dependent. At DPPC liquid-crystalline phase average headgroup orientation angle *< ω >*= 69.36 degrees, which agrees with median angle *< ω >* between N and P dipole and normal vector obtained in MD simulation (*< ω >*= 68 degrees). The difference of *< ω >* between liquid-crystalline and gel-crystalline phase is a consequence of a different values of area per lipid *a*_0_ in gel-crystalline phase and liquid-crystalline phase.

## 4. Discussion and Conclusions

**Figure 6:**
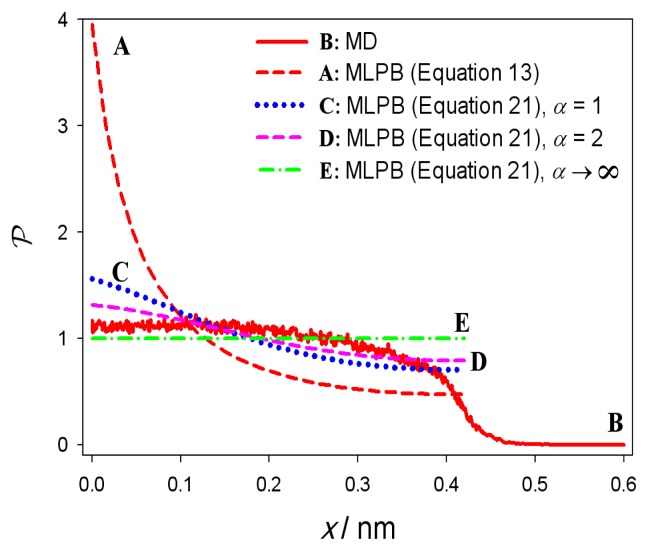
Probability density *℘*(*x*) that the positive charge of the lipid dipolar headgroup (see also Figure 2) is located at the distance *x* from the negatively charged surface calculated from MLPB model (A,C,D,E) and obtained from MD simulations (B). The values of MLPB model parameters are the same as in Figure 4.

The comparison between the probability density *℘*(*x*) calculated within MLPB model (Equation 13) and *℘*(*x*) obtained in MD simulations, can be seen on Figure 6. In MLPB model the function *℘*(*x*) is steeply increasing in the vicinity of *x* = 0 plane (Figure 6, plot A) which is a consequence of the exponential Boltzmann factor in the function *℘*(*x*) (Equation 13). This result is clearly not in accordance with MD simulation (Figure 6, plot B), where the function *℘*(*x*) is saturated. The discrepancy between the predictions of MLPB model and MD simulations arises due to the fact that the finite volumes of lipid headgroups and finite volumes of ions and water molecules are not considered in MLPB model. Taking into account the finite volume of lipid headgroups within the lattice statistics approach (see also [13,24]) yields for probability density that the positive charge of the dipolar lipid headgroup is located at certain distance *x* from the *x* = 0 surface in the form :

(21) $$P(x)=\Lambda \frac{\alpha \;\text{exp}(-e_{0}\varphi (x)\beta )}{\alpha \;\text{exp}(-e_{0}\varphi (x)\beta )+1}$$

where Λ is determined from normalization Equation 14. The parameter *α* is equal to the ratio between the average volume of the positively charged parts of dipolar (zwitterionic) headgroups and the average volume of the salt solution in the headgroup region. Equation 21 predicts the saturation of the probability density function *℘*(*x*), corresponding to the close packing of the lipid headgroups in accordance with the results of MD simulations. Figure 6 thus shows the dependence of *℘*(*x*) calculated by using Equation 21 for the values of the parameter *α*. It can be seen that taking into account the finite volume of lipid headgroups leads to better agreement between the predicted *℘*(*x*) dependencies within MLPB model and MD simulations. In the limit of *α* → ∞ (when all lattice sites are occupied by the headgroups) the probability density function *℘*(*x*) becomes constant as expected. On the other hand, in the limit of small values of *α* (*i.e*., negligible volume of the headgroups) the probability density *℘*(*x*), calculated by using Equation 21, approaches to the probability denisty *℘*(*x*) determined by Equation 13 (curve A in Figure 6).

Although the relative permittivity *ɛ_r_* is usually considered as a constant, it is shown in present paper that *ɛ_r_* can considerably change within the dipolar (zwitterionic) lipid headgroup region. Consequently, the electric potential in this region is substantially decreased.

To conclude, our model shows that the effect of decreasing potential and permittivity has an impact only in the headgroup region of dipolar zwitterionic lipids and its close vicinity. The average orientation angle of the zwitterionic lipid headgroup dipole (*< ω >*) predicted within the presented MLPB model is comparable with the results obtained in MD simulation.

## A. Derivation of Equations for Matlab

### A.1. Variant A

Equation 16 can be rewritten as :

(22) $$\frac{{\text{d}}^{2}\psi (x)}{\text{d}x^{2}}=\kappa^{2}(x)\;\text{sinh}(\psi (x))-\frac{\kappa^{2}(x)\;\text{exp}(-\psi (x))}{2n_{0}a_{0}\int_{0}^{D}\text{exp}(-\psi (x))\text{d}x}-\frac{1}{{ɛ}_{r}(x)}\frac{\text{d}{ɛ}_{r}(x)}{\text{d}x}\frac{\text{d}\psi (x)}{\text{d}x}$$

where *ψ*(x) is reduced electric potential :

(23) $$\psi (x)=e_{0}\beta \varphi (x)$$

and

(24) $$\kappa^{2}(x)=\frac{2e_{0}^{2}n_{0}\beta }{{ɛ}_{0}{ɛ}_{r}(x)}$$

The boundary conditions (Equations 17–20) can be rewritten as :

(25) $$\frac{\text{d}\psi (x)}{\text{d}x}(x=0)=-\frac{e_{0}\beta \sigma }{{ɛ}_{0}{ɛ}_{r}(x=0)}$$

(26) $$\frac{\text{d}\psi }{\text{d}x}(x\to \infty )=0$$

(27) $$\psi (x=D_{-})=\psi (x=D_{+})$$

(28) $$\frac{\text{d}\psi }{\text{d}x}(x=D_{-})=\frac{\text{d}\psi }{\text{d}x}(x=D_{+})$$

Equation 22 was solved in Matlab using the standard function for the boundary value problems (bvp4c). The second part of the right hand side of Equation 22 is defined only within headgroup region, therefore it is equal to zero for *x > D*. The value of *ɛ**_r_*(*x*) (Equation 6) and the integral ∫ exp(−*ψ*(*x*))d*x* and d*ɛ**_r_*(*x*)*/*d*x* in Equation 22 were calculated outside of bvp4c function. The d*ɛ**_r_*(*x*)*/*d*x* was derived from Equation 6, where the Langevin function *ℒ*(*x*) was expanded for small values of electric field into Taylor series up to the cubic term *ℒ*(*x*) ≈ *x/*3 − *x*^3^*/*45. Considering $\tilde{E}(x)=\left|\frac{\text{d}\psi (x)}{\text{d}x}\right|$, can be written :

(29) $${ɛ}_{r}(x)=K_{1}+\frac{K_{2}}{45}\left(15-{\left(K_{3}\tilde{E}(x)\right)}^{2}\right)$$

(30) $$\frac{\text{d}{ɛ}_{r}(x)}{\text{d}x}=-\frac{2K_{2}K_{3}^{2}}{45}\frac{\text{d}\psi (x)}{\text{d}x}\frac{{\text{d}}^{2}\psi (x)}{\text{d}x^{2}}$$

where the constants are :

(31) $${\text{K}}_{1}=n^{2}$$

(32) $${\text{K}}_{2}=\frac{3}{2}{\left(\frac{2+n^{2}}{3}\right)}^{2}\frac{n_{0w}p_{0}^{2}\beta }{{ɛ}_{0}}$$

(33) $${\text{K}}_{3}=\frac{\gamma p_{0}}{e_{0}}$$

For high values of electric field the *ɛ**_r_*(*x*) and the d*ɛ**_r_*(*x*)*/*d*x* are derived from Equation 6 with non-expanded Langevin function :

(34) $${ɛ}_{r}(x)=K_{1}+K_{2}\left(\frac{(K_{3}\tilde{E}(x))\;\text{coth}(K_{3}\tilde{E}(x))-1}{{\left(K_{3}\tilde{E}(x)\right)}^{2}}\right)$$

(35) $$\frac{\text{d}{ɛ}_{r}(x)}{\text{d}x}=\frac{K_{2}}{\frac{\text{d}\psi (x)}{\text{d}x}}\left(-\frac{1}{{\text{sinh}}^{2}(K_{3}\tilde{E}(x))}-\frac{(K_{3}\tilde{E}(x))\;\text{coth}(K_{3}\tilde{E}(x))-2}{{\left(K_{3}\tilde{E}(x)\right)}^{2}}\right)\;\left(\frac{{\text{d}}^{2}\psi (x)}{\text{d}x^{2}}\right)$$

where constants are the same as above (Equations 31–33).

### A.2. Variant B

By inserting the Equation 6 and its derivative into Equation 8, the Equation 8 can be rewritten as :

(36) $$\frac{{\text{d}}^{2}\psi (x)}{\text{d}x^{2}}=\frac{\frac{2{e_{0}}^{2}n_{0}}{{ɛ}_{0}\beta }\;\text{sinh}(\psi (x))-\frac{{e_{0}}^{2}\;\text{exp}(-\psi (x))}{a_{0}{ɛ}_{0}\beta \int_{0}^{D}\text{exp}(-\psi (x))\text{d}x}}{\left[K_{2}\left(\frac{1}{{\left(K_{3}\tilde{E}(x)\right)}^{2}}-\frac{1}{{\text{sinh}}^{2}(K_{3}\tilde{E}(x))}\right)+K_{1}\right]}$$

where the constants *K*1, *K*2, *K*3 are defined as above (Equations 31–33). Equation 36 was solved in Matlab using standard function for boundary value problem (bvp4c) with multipoint boundary values (Equations 25–28). The value of the integral ∫ exp(−*ψ*(*x*))d*x* in Equation 36 was calculated in iteration process outside of bvp4c function.

## B. Average Orientation of Lipid Head-Groups

From Equations (13 and 15) it follows:

(37) $$P(x)=\frac{D\;\text{exp}(-\psi (x))}{\int_{0}^{D}\text{exp}(-\psi (x))\text{d}x}$$

Average orientation angle of the headgroup dipole (*< ω >*) can be then written as normalized distribution function:

(38) $$<\omega >\frac{\int_{0}^{D}\omega P(x)\text{d}x}{\int_{0}^{D}P(x)\text{d}x}$$

where the dipole orientation angle is (see also Figure 2) :

(39) $$\omega =\text{arccos}\left(\frac{x}{D}\right)$$
